# The Correlation between Motor Skill Proficiency and Academic Performance in High School Students

**DOI:** 10.3390/bs14070592

**Published:** 2024-07-12

**Authors:** Domingo Jesús Ramos-Campo, Vicente Javier Clemente-Suárez

**Affiliations:** 1 LFE Research Group, Department of Health and Human Performance, Faculty of Physical Activity and Sport Science (INEF), Universidad Politécnica de Madrid, 28040 Madrid, Spain; 2 Faculty of Sports Sciences, Universidad Europea de Madrid, Tajo Street, s/n, 28670 Madrid, Spain; vctxente@yahoo.es; 3 Grupo de Investigación en Cultura, Educación y Sociedad, Universidad de la Costa, Barranquilla 080002, Colombia

**Keywords:** motor skills, academic performance, physical education, functional movement screen, high school students, cognitive development

## Abstract

The intricate relationship between physical health and cognitive development has been a focal point of multidisciplinary research, suggesting that motor skill proficiency could influence academic outcomes. This study aimed to investigate the correlation between motor control, mobility, stability—measured by the Functional Movement Screen (FMS)—and academic performance among high school students. Conducted with 201 participants from a public high school in Spain, this descriptive non-experimental research divided students into high and low academic performance groups based on their average grade scores, with the median used as the criterion for distinction. The FMS was utilized to assess fundamental motor skills, and academic performance was verified across mathematics, language, science, and physical education subjects. The findings revealed a significant positive relationship between the FMS scores and academic achievement (*r* = 0.691; *p* < 0.001), indicating that students with higher motor skill proficiency had higher academic achievement. This correlation persisted across the various subjects, highlighting the potential benefits of integrating physical education and motor skill development into educational strategies. The study’s results suggest that educational policies should advocate for comprehensive physical education programs to foster both physical well-being and academic improvement, thereby promoting a holistic educational model that enhances student performance across disciplines.

## 1. Introduction

In recent years, the intricate link between physical well-being and academic achievement has garnered attention across multidisciplinary fields, blending insights from education, psychology, and health sciences [1,2,3,4]. There is growing evidence that physical activity plays a crucial role in cognitive development and educational outcomes, as suggested by recent studies [5]. However, the specific impact of motor control, mobility, and stability on academic success is less clear. This research aims to delve into how these essential motor skills correlate with academic performance in high school students, using the Functional Movement Screen (FMS) as a primary assessment tool. The FMS evaluates the fundamental movement patterns essential for physical activity. The FMS was chosen because it identifies dysfunction in movements requiring an interplay of cognitive, perceptual, proprioceptive, and motor functions, involving muscular strength, endurance, flexibility, mobility, coordination, and balance [6,7]. Recent studies have shown that motor skills are closely related to cognitive functions and academic performance, highlighting the importance of physical education in school curricula [8,9,10,11]. While our study focuses on academic performance rather than direct cognitive evaluation, it was hypothesized that improved motor skills, as measured by the FMS, can support better academic outcomes. Recent research supports this hypothesis by demonstrating the link between motor skills and academic performance [8,9,10,11]. For instance, a previous systematic review provides evidence that motor skills, including coordination and balance, are significantly associated with academic performance in subjects such as mathematics and reading [12]. The study suggests that the development of motor skills can enhance processes that support learning. Recent interventions aimed at improving motor control and stability have shown improvements in academic outcomes, indicating a potential pathway for educational enhancements through physical education programs. The findings support the notion that physical well-being is intimately linked to academic achievement, reinforcing the importance of incorporating motor skill development into educational strategies [13,14,15].

Continuing from the premise that physical well-being is intertwined with academic success, it becomes imperative to scrutinize the role of motor abilities within this framework. Previous research has underscored the importance of motor skills, revealing a correlation between these physical attributes and cognitive achievements in educational settings [16]. For instance, studies have found that motor skills, such as coordination and balance, not only enhance physical health but also bolster the cognitive functions critical for learning, including attention span and memory retention [9]. This link suggests that motor control, mobility, and stability might serve as foundational components that support academic pursuits by fostering an environment conducive to cognitive engagement and processing. Moreover, interventions focusing on the development of motor skills have been shown to yield positive outcomes in academic performance, hinting at the potential benefits of integrating physical education with educational curricula to promote holistic student development [16]. In this line, the impact of overweight and obesity on children’s academic performance underscores the urgent need for programs that promote healthy habits encompassing both physical activity and nutrition. Such initiatives aim to significantly enhance children’s overall health, psychological well-being, and cognitive and motor skill development, thereby improving their academic outcomes [1].

In alignment with previous research, our findings indicate a significant positive correlation between motor skill proficiency and academic performance. Haapala [9] and Lubans et al. [8] have demonstrated this relationship in younger children, suggesting that the cognitive benefits of physical activity and motor skill development are applicable across different age groups. Other research supported these findings, showing improvements in executive functions in children aged 3–5 years following physical activity interventions. These studies provide substantial evidence supporting the impact of motor skills on academic performance, reinforcing the relevance and applicability of our study’s outcomes across various age ranges [14,15,16].

Given the evidence linking physical capabilities with cognitive functions and academic achievements, this study aimed to examine the relationship between motor control, mobility, stability, and academic performance in high school students. It was hypothesized that higher scores in motor control, mobility, and stability, as measured by the Functional Movement Screen, would be significantly correlated with superior academic performance in high school students. The utilization of the Functional Movement Screen (FMS) as a measure provides a standardized method to assess these motor skills, offering insights into how foundational physical competencies might influence educational outcomes [17,18,19]. Previous studies have demonstrated the reliability and validity of the FMS in assessing movement patterns and their implications for overall physical health and performance [20,21]. This descriptive, non-experimental research underscores the significance of physical education in the academic curriculum, potentially guiding policy changes to incorporate comprehensive physical development programs in schools. Such an approach aligns with a growing body of research advocating for the integration of physical and cognitive development strategies to enhance student performance across various disciplines [1,5]. The activities evaluated with the FMS are hypothesized to improve overall academic performance by enhancing essential motor skills that support the cognitive functions crucial for learning [3,4]. Such an approach aligns with the growing body of research advocating for the integration of physical and cognitive development strategies to enhance student performance across various disciplines [5,22,23]. The activities evaluated with the FMS are hypothesized to improve overall academic performance by enhancing essential motor skills that support the cognitive functions crucial for learning [5,23,24].

## 2. Materials and Methods

### 2.1. Design

This descriptive, cross-sectional comparative study with a non-experimental design examined the relationship between motor control, mobility, stability, measured by the Functional Movement Screen, and academic performance in high school students. The independent variable was related to academic performance, while the dependent variable concerned performance on a test of motor control skills.

### 2.2. Participants

Two hundred and one high school students voluntarily participated in the current study, with an average age of 13.9 ± 1.6 years (range: 12–16 years), a weight of 61.1 ± 13.1 kg, a height of 167.4 ± 8.6 cm, and a sex distribution of 56.3% boys (n = 113) and 43.7% girls (n = 88). All participants were students at a public high school in Murcia, Spain. They participated in physical activity three days per week, with each session lasting 60 min. To accomplish the primary objective of this research, the participants were stratified into two groups of equal size based on their academic performance (50th percentile) in terms of their average grade score at the end of the academic year, coinciding with the timing of the Functional Movement Screen (FMS)assessment. The low academic performance group (LAPG: n = 101) comprised scores between 3.25 and 6.5 out of 10, while the high academic performance group (HAPG: n = 100) included scores between 6.5 and 9.5 out of 10. Participants for this study were recruited using convenience sampling. 

The study objectives were communicated to fathers, mothers, the fathers’ association, school management team, and teachers. Each mother, father, or legal guardian of the students received a personal explanation of this study, along with an informed consent form and study information sheet. Students whose consent forms, signed by their parent or legal guardian, were submitted to the evaluators were included in the analysis. Approximately 46% of the school students agreed to participate in the study. All participants, including parents/legal guardians and teachers, were briefed on the experimental procedures and informed of their right to withdraw from this study at any time. Prior to commencing this research, all participants were required to complete an informed consent form, adhering to the principles outlined in the Declaration of Helsinki (revised in Brazil, 2013). Data collection was conducted anonymously, and the study protocol received approval from the European University of Madrid Ethical Committee (CIPI/18/074).

### 2.3. Procedures

All assessments were conducted in a single testing session during the final week of the academic year. The session commenced with a standardized 10 min warm-up, encompassing low-intensity running, joint mobility exercises, and dynamic stretching. Subsequently, participants underwent the Functional Movement Screen (FMS) test [25,26]. The FMS comprises seven tasks, evaluating fundamental movement patterns requiring a balance of joint mobility and neuromuscular control: (i) deep squat (a dowel is placed over the head with the arms outstretched and the individual squats as low as possible); (ii) hurdle step (a dowel is placed across the shoulders and the individual steps over a hurdle placed directly in front of them); (iii) the in-line lunge (with the feet aligned and a dowel contacting the head, back, and sacrum, the individual performs a split squat); (iv) shoulder mobility test (the individuals attempt to touch their fists together behind their back by internal and external shoulder rotation); (v) active straight leg raise (while lying supine with their head on the ground, the individuals actively raise 1 leg as high as possible); (vi) trunk stability push-up (the individuals perform a push-up with their hands shoulder width apart); and (vii) rotary stability test (the individuals assume a quadruped position and attempts to touch their knee and elbow, first on the same side of the body and then on the opposite side). Additionally, three clearing tests were conducted to check for pain during shoulder internal rotation/flexion and end-range spinal flexion and extension. Each of the seven movements was scored on a scale from 0 to 3, with a maximum achievable score of 21. Three attempts were made for each task and were scored by two investigators with two years of FMS experience, demonstrating inter-investigator reliability with an intraclass correlation coefficient of 0.876. Previous research supports the use of two evaluators for reliable assessments, demonstrating that the FMS test has good inter- and intrareproducibility with two evaluators [19]. The total FMS score, ranging from 0 to 21, was utilized for analysis.

Furthermore, the academic performance analysis included retrieving end-of-year scores for the average, mathematics, language, science, and physical education. The official grades issued by the educational institute at the end of the academic year were used. Academic achievement scores could vary from 0 to 10, with an interval of 0.1. In this school system, the minimum passing score is 5.0 points.

### 2.4. Statistical Analysis

Statistical analysis was performed using Jamovi 2.3.28 statistical software for Windows (The Jamovi project, Sydney, Australia). Descriptive statistics, encompassing mean and standard deviation, were computed. Before using parametric tests, normality and homoscedasticity assumptions were assessed through the Kolmogorov–Smirnov test. An independent *t*-test was used to determine group differences in the analyzed variables, while the Pearson correlation was calculated to assess the relationship between FMS and academic performance variables. A significance level of *p* ≤ 0.05 was chosen for all procedures.

## 3. Results

All the variables followed a normal distribution. Table 1 presents the differences in the Functional Movement Screen (FMS) scores and the academic performance of the low academic performance (LAP) and high academic performance (HAP) groups. The HAP group exhibited significantly elevated FMS values (16.4 ± 2.1 points) compared to the LAP group (13.3 ± 2.2 points; *p* < 0.001; *d* = −1.46). Furthermore, as expected, the HAP group demonstrated significantly higher average scores in all subjects (mathematics, language, science, and physical education) compared to the LAP group.

**Figure 1:**
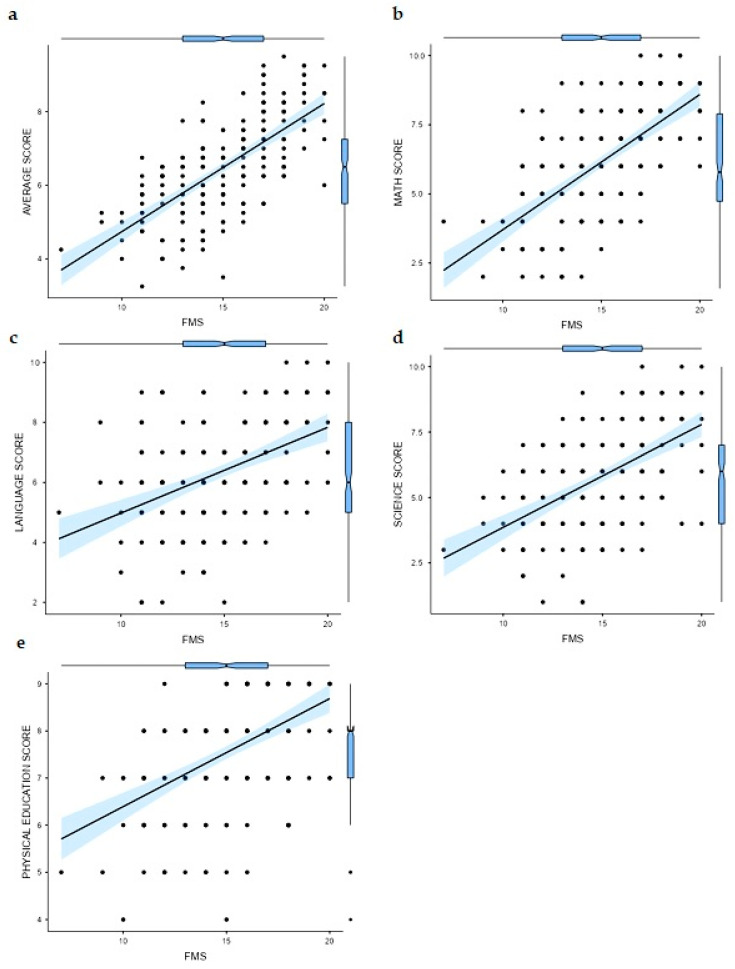
Correlation results between Functional Movement Screen values and (**a**) average score; (**b**) mathematics score; (**c**) language score; (**d**) sciences score; (**e**) physical education score.

Regarding the correlation results, a significant positive relationship was found between the FMS values and average score (*r* = 0.691; *p* < 0.001) (Figure 1a). Additionally, a significantly positive relationship was observed between the FMS values and the rest of the matter analyzed (mathematics score (*r* = 0.652; *p* < 0.001) (Figure 1b), language score (*r* = 0.438; *p* < 0.001) (Figure 1c), sciences score (*r* = 0.536; *p* < 0.001) (Figure 1d), and physical education score (*r* = 0.510; *p* < 0.001) (Figure 1e)). 

## 4. Discussion

The high academic performance (HAP) group showed significantly higher FMS scores than the low academic performance (LAP) group, indicating a positive correlation between motor skill proficiency and academic success. These results are consistent with previous findings that highlight the benefits of physical activity on cognitive functions and academic performance [27]. Recent evidence from the Aadland group further supports this, demonstrating improvements in cognitive processes through neuro-psychological tests following physical activity interventions [28]. Also, other studies reinforced the idea that regular physical activity can lead to improvements in attention, memory, and executive control, which are essential components of academic success, highlighting how students participating in consistent physical activity displayed better cognitive outcomes, including in areas of academic performance such as mathematics and reading scores [27]. A study of the biological underpinnings of this relationship suggested that physical activity may improve brain health and cognitive function through enhanced neuroplasticity, increased brain-derived neurotrophic factor (BDNF) levels, and improved vascular health [29]. This biological perspective offers a physiological explanation for the observed correlation between motor skills and academic performance, suggesting that the benefits of physical fitness extend beyond the gym or playground and into the classroom [30,31]. Physical activity has been shown to activate the neurobiological processes that form the basis of neuroplasticity, including the upregulation of neurotrophic factors, increased synaptic plasticity, and enhanced neurogenesis. This is supported by extensive research indicating that physical exercise enhances brain function and promotes neuroplasticity across the lifespan [32,33,34,35].

While our study focused on academic performance, it is essential to discuss how specific aspects of the FMS test, such as motor control, stability, and mobility, can be analyzed from cognitive dimensions. For instance, the execution of the deep squat movement in the FMS test requires a complex sequence of actions involving various brain regions. Planning the movement sequence involves the most anterior cerebral cortex, while the dorsolateral prefrontal cortex is responsible for executing the plan through the primary motor cortex. The orbital frontal cortex exerts control by inhibiting unnecessary movements, and the prefrontal cortex’s working memory retains the instructions. Additionally, sensory perception and subcortical structures contribute to precision, modulation, and balance. Linking these cognitive processes to academic performance, it is plausible that students who perform well in the FMS tests demonstrate better executive functions, which are crucial for subjects requiring complex problem-solving and critical thinking, such as mathematics and science. This aligns with research showing that physical activity enhances the executive functions, including planning, attention, and memory retention, which are directly applicable to academic tasks [36,37,38,39,40].

The notable disparity in average scores across subjects (LAP: 5.4 ± 0.7; HAP: 7.5 ± 0.8 points), including mathematics, language, science, and physical education, between the HAP and LAP groups highlights significant academic differences and underscores the multifaceted influence of physical well-being on cognitive functions [41]. While these differences suggest that higher motor skill proficiency is associated with better academic performance, they do not directly imply that physical well-being influences cognition. The observed correlations indicate that students with better-developed motor skills tend to achieve higher academic scores, underscoring the potential benefits of integrating physical education programs to support academic success. However, it is important to note that this study did not establish a causal relationship between physical fitness and cognitive function. This result is in line with a previous meta-analysis revealing that physical activity interventions have a significant positive effect on children’s cognitive functioning, suggesting that improvements in physical fitness can enhance aspects of children’s mental capabilities crucial for learning [16]. Specifically, in the mathematics and reading areas, it was found how physical activity interventions in schools also have a significant positive impact, confirming the integral role of physical health in educational success, advocating for the incorporation of physical activity into daily school routines to optimize students’ academic outcomes [42]. 

Furthermore, the positive correlations found between the FMS values and the individual academic subjects (math, language, science, and physical education scores) highlight the role of fundamental movement skills in academic achievement. This is consistent with the findings of Haapala (2013) [9], who argued that motor skills, especially balance and coordination, are closely linked to executive functions and academic performance in children. The similarity in trends across various studies emphasizes the potential benefits of integrating physical education and motor skill development into academic curricula to foster both physical and cognitive development. For instance, it was demonstrated that exercise can enhance children’s mental processing speed, attention, and executive functions, which are critical for academic performance [36]. Similarly, it was shown how interventions designed to improve executive functions, which are closely related to physical activity, could have a positive impact on educational outcomes [37]. These correlations between the FMS scores and the various academic subject scores suggest that fundamental motor skills contribute to the cognitive functions that are essential for learning and academic success [43,44]. Moreover, the relationship between physical fitness and academic performance is further supported by other authors, who found that physically fit children tend to have larger hippocampal volumes, leading to better memory performance [45]. The neurological evidence underscores the role of physical fitness in enhancing brain structures associated with memory, an essential skill for academic learning.

However, the comparison of these findings with previous studies also invites consideration of the varying methodologies and demographic factors that may influence the strength of the observed relationships. For instance, differences in age, socioeconomic status, and educational system could account for the variations in the significance and magnitude of the correlations reported across studies. It is also worth noting that while the present study provides valuable insights into the association between motor skills and academic performance, the cross-sectional design limits the ability to infer causality.

Finally, a higher correlation was observed between the FMS scores and mathematics performance compared to other subjects. This can be attributed to the cognitive demands of mathematics, which requires high levels of concentration, problem-solving skills, and the ability to manage complex tasks. Physical fitness and motor skill proficiency, as measured by the FMS, enhance cognitive functions such as working memory, attention, and executive function, which are essential for mathematical success [46,47,48]. Regular physical activity boosts brain health, increases neuroplasticity, and enhances neural efficiency, contributing to improved mathematical problem-solving abilities [49]. Additionally, students with higher motor skill proficiency may exhibit better self-regulation and discipline, traits that are particularly beneficial in the structured learning environment of mathematics [50].

### Limitations, Practical Application, and Future Research Directions

The findings of this study underscore the importance of integrating physical education and targeted motor skill development programs into educational curricula to potentially enhance academic performance. Recommendations are made for schools and educational policymakers to adopt comprehensive physical fitness assessments, such as the Functional Movement Screen, as routine evaluations for students. Addressing deficits in motor control, mobility, and stability early on through tailored physical activity programs may improve physical health and support the cognitive functions essential for academic success.

However, it is essential to acknowledge several limitations. The cross-sectional design, while beneficial for identifying correlations, precludes establishing causal relationships. Additionally, reliance solely on the FMS may not fully capture the breadth of physical abilities influencing academic outcomes. This study’s reliance on a single educational institution limits the generalizability of the findings. Furthermore, potential confounding factors such as socioeconomic status, classroom environment, and individual cognitive abilities could influence observed relationships.

Future research directions should prioritize longitudinal designs to clarify the directionality of the relationships between motor skills and academic performance. Including a wider array of physical assessments and diverse demographic samples would offer a more comprehensive understanding of the interplay between physical and cognitive domains. Investigating specific interventions aimed at improving motor skills and assessing their direct impact on academic outcomes could provide actionable insights for educators and policymakers. Addressing these limitations and exploring future research avenues will advance the field toward evidence-based interventions that optimize the role of physical education in promoting academic achievement.

## 5. Conclusions

The findings of the present research revealed a significant positive correlation indicating that students with higher levels of motor control, mobility, and stability tend to achieve better academic outcomes across various subjects. These results underscore the importance of integrating physical education and targeted motor skill development into educational curricula to support both physical and cognitive development. Specifically, the Functional Movement Screen (FMS) activities, which assess fundamental movement patterns, appear to positively influence academic performance in subjects such as mathematics, language, science, and physical education. For example, improved coordination and balance, as evaluated through the FMS, could enhance students’ performance in mathematics and science by supporting better focus and problem-solving skills. However, it is essential to note that a direct relationship between motor skills and academic performance has not been conclusively established due to the potential presence of extraneous variables. Factors such as socioeconomic status, classroom environment, and individual cognitive abilities could also play a significant role in academic outcomes. Future research should aim to control for these variables to better isolate the effects of motor skill proficiency on academic performance. By highlighting the specific benefits of the FMS activities and acknowledging the limitations of our study, we aim to provide a more nuanced understanding of the relationship between physical and academic performance.

## Figures and Tables

**Table 1:** Differences in Functional Movement Screen score and academic performance between low academic performance and high academic performance groups.

Outcome	LAP (n = 101)	HAP (n = 100)	T	*p* Value	Mean Difference	Effect Size (*d*)
Average Score	5.4 ± 0.7	7.5 ± 0.8	−19.68	<0.001	−2.15	−2.78
Mathematics Score	4.7 ± 1.4	7.5 ± 1.3	−15.13	<0.001	−2.88	−2.13
Language Score	5.5 ± 1.5	7.3 ± 1.4	−9.06	<0.001	−1.84	−1.28
Science Score	4.4 ± 1.3	7.2 ± 1.4	−14.38	<0.001	−2.74	−2.03
Physical Education Score	7.0 ± 1.2	8.1 ± 0.9	−7.81	<0.001	−1.14	−1.10
FMS Score	13.3 ± 2.2	16.4 ± 2.1	−10.36	<0.001	−3.10	−1.46

FMS: Functional Movement Screen; LAP: low academic performance; HAP: high academic performance.

## Data Availability

All data are in the article.
